# Tracheoesophageal fistula associated with bevacizumab after thoracic radiotherapy in non-small cell lung cancer: A case report

**DOI:** 10.1097/MD.0000000000019878

**Published:** 2020-04-24

**Authors:** Tao Zhang, Yin Yang, Guowei Cheng, Ping Chen, Nan Bi

**Affiliations:** a Department of Radiation Oncology, National Cancer Center/National Clinical Research Center for Cancer/Cancer Hospital, Chinese Academy of Medical Science and Peking Union Medical College; b Department of Radiation Oncology, Cancer Hospital of Huan Xing Chao Yang District Beijing, Beijing, China.

**Keywords:** bevacizumab, radiotherapy, tracheoesophageal fistula

## Abstract

**Introduction:**

Tracheoesophageal Fistula (TF) is a rare complication of Bevacizumab. Thoracic radiotherapy may be a contributing factor to TF formation. To the best of our knowledge, we report the first case of Chinese patient with non-small cell lung cancer (NSCLC) who developed TF after completion of chemotherapy with bevacizumab and thoracic radiotherapy.

**Patient concerns:**

A 54-year-old male patient was diagnosed with NSCLC. He received definitive thoracic radiotherapy with concurrent pemetrexed and cisplatin chemotherapy. Two months after the treatment, the disease progressed with enlargement of right inguinal lymph node and chemotherapy of docetaxel, carboplatin and bevacizumab was administrated. Eighteen days after 4 cycles, the patient presented a sudden onset of acute cough after drinking.

**Diagnosis:**

Esophageal barium swallow revealed a TF. Gastroscopy confirmed a fistula in the esophagus.

**Interventions:**

A jejunal feeding tube was placed for nutrition for a month. After that a covered esophageal stent was placed in the esophagus.

**Outcomes:**

At the 6-month follow-up visit, the patient recovered well and had not developed any complication related to the stent placement.

**Conclusion:**

TF is a rare but life-threatening complication of bevacizumab. Careful observation is imperative for those patients who are administered bevacizumab, particularly in patients treated previously with thoracic radiotherapy.

## 1 Introduction

Bevacizumab, a chimeric hominization immunoglobulin G1 monoclonal antibody against vascular endothelial growth factor, can prevent the development of new blood vessels needed for tumor cells to grow. Bevacizumab, in combination with carboplatin and paclitaxel, is approved by the US Food and Drug Administration for the first-line treatment of patients with advanced/metastatic recurrent non–squamous non-small cell lung cancer (NSCLC).^[1]^ NCCN Guidelines^[2]^ recommends bevacizumab combining chemotherapy as initial systemic therapy options for advanced or metastatic non–squamous NSCLC. Tracheoesophageal fistula (TF), a rare but severe complication of bevacizumab, had been reported in patients from different countries. However, there have been no such reports of patients from China. We describe a 54-year-old male with history of thoracic radiotherapy who developed a TF 2 weeks after completion of 4 cycles of chemotherapy with bevacizumab.

## 2 Case report

**Figure 1:**
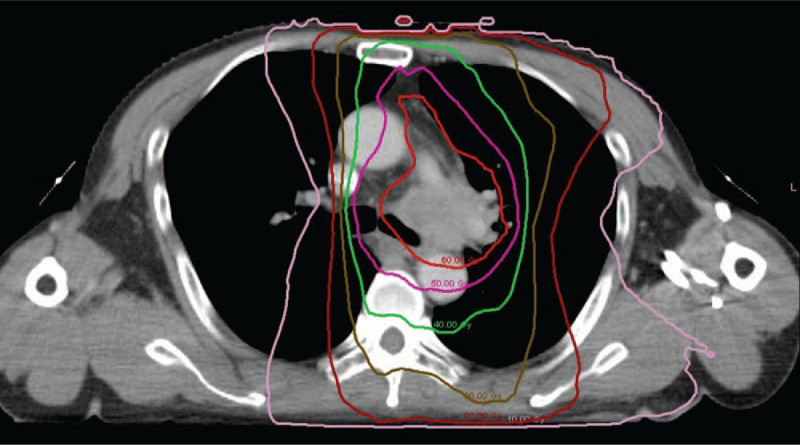
Isodose lines showing radiation dose distribution in a 54-yr-old male with stage IIIB non-small cell lung cancer.

A 54-year-old male was referred to local hospital with cough and expectoration in April 2018. Chest computed tomography (CT) revealed a mass in the left of the lung, with multiple mediastinal lymph node metastases (4L, 5 and 6 region). Endobronchial ultrasound-guided transbronchial needle aspiration result was consistent with adenocarcinoma. Staging evaluation in Cancer Hospital, Chinese Academy of Medical Sciences (Beijing, China) with CT, positron emission tomography- CT, and magnetic resonance imaging of the brain conformed stage IIIB (T3N2M0) according to the UICC 7th edition TNM classification. The patient was given concurrent chemotherapy, consisting of 500 mg/m^2^ pemetrexed and 75 mg/m^2^ cisplatin on day 1 every 3 weeks for 2 cycles, and definitive thoracic radiotherapy of volumetric modulated arc therapy (95%PGTV 60.2Gy in 28 fractions. 95%GTV 50.4Gy in 28 fractions). The isodose lines of radiation dose distribution are showed in Figure 1. A CT scan of the chest indicated a partial response. Grade I esophageal toxicity, Grade II gastrointestinal toxicity, Grade I dermatological toxicity and Grade I myelotoxicity (Common Terminology Criteria for Adverse Events Version 4.0; CTCAE v4.0) were the side effects of the concurrent chemoradiotherapy.

However, 2 months after completing concurrent chemoradiotherapy, he developed progressive disease with enlargement of right inguinal lymph node. Biopsy of the inguinal lymph node was consistent with adenocarcinoma and the patient was administered docetaxel 75 mg/m^2^ on day 1, carboplatin AUC 5 on day 2 plus bevacizumab 7.5 mg/kg on day 1 once every 3 weeks.

**Figure 2:**
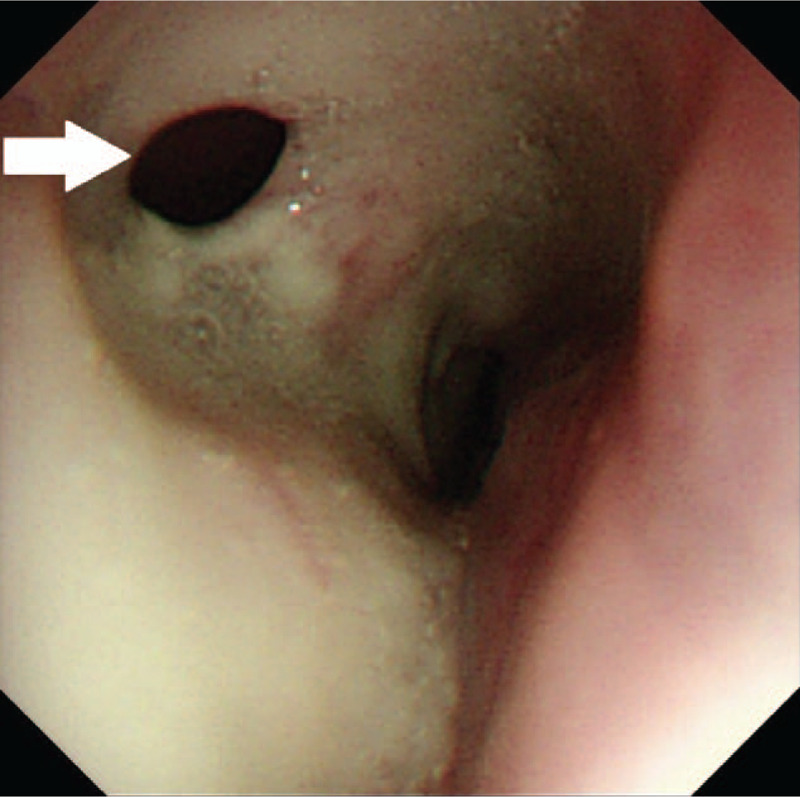
A tracheoesophageal fistula (white arrow) was observed by means of gastroscopy.

**Figure 3:**
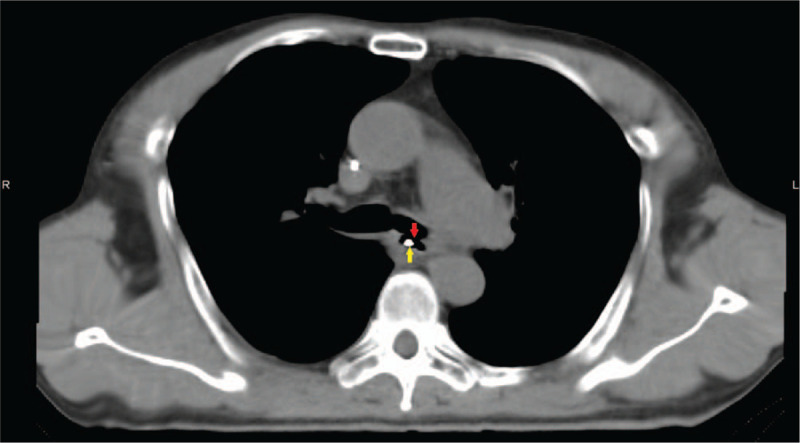
A chest computed tomography scan showed a tracheoesophageal fistula (red arrow) and jejunal feeding tube (yellow arrow).

**Figure 4:**
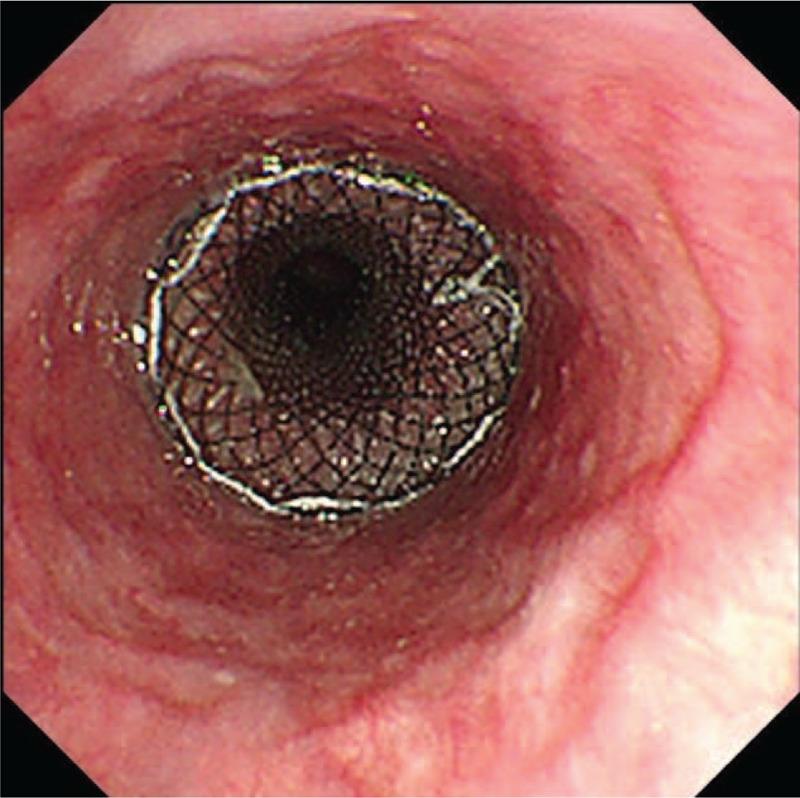
A covered esophageal stent in the esophagus.

Eighteen days after 4 cycles, the patient presented a sudden onset of acute cough after drinking. Esophageal Barium meal revealed a TF. Gastroscopy showed an esophageal mucosa erosion to be 28 to 36 cm from the nostrils, in which a deep hole was observed (Fig. 2). Then a jejunal feeding tube was placed for nutrition (Fig. 3). Forty days after the placement, the patients suffered from lung infections by an unclosed TF and a covered esophageal stent was placed in the esophagus (Fig. 4). The TF was managed with stents. Until now, 6 months after the event of TF, the patient was still in follow-up care without any complication related to the stent placement.

## 3 Discussion

Bevacizumab, an anti-vascular endothelial growth factor receptor antibody, has been demonstrated to have activity against advanced or recurrent non-squamous NSCLC. A phase III study demonstrated that combination of Bevacizumab and chemotherapy of carboplatin and docetaxel can significantly improve patients’ overall survival, progression free survival, and partial response rate.^[3]^ However, bevacizumab displays several side effects such as thromboembolism, hypertension, bleeding, proteinuria.^[4]^ Bevacizumab related TF is a rare but life-threatening complication which has been reported in the treatment of lung cancer. In a phase I/II trial of bevacizumab plus erlotinib for pancreatic cancer treatment, TF developed in one (2%) patients.^[5]^ Several literatures from different countries including Japan,^[6]^ American,^[7]^ Germany^[8]^ have reported the TF occurred after administering of bevacizumab. However, no such report of Chinese is available at this time. This is the first case of TF after administering of radiotherapy and bevacizumab for Chinese patient with NSCLC.

The mechanism of TF formation with bevacizumab is unknown, but it is hypothesized that impaired angiogenesis leads to delayed wound healing. The predisposing factors suggested in previous reports for TF are tumor location,^[6]^ tracheoesophageal injury due to instrumentation, a history of thoracic radiotherapy.^[9]^

Among all reports of TF, the time between the TF and radiotherapy in NSCLC is different, the shortest time is 2 months after the completion of radiotherapy^[10]^ and the longest time is 21 months.^[7]^ In our case, the patient suffered TF 5 months after the completion of radiotherapy. Acute esophageal toxicity is a common side effect of radiotherapy and predisposition for late TF. In a phase II clinical trial,^[9]^ all two patients of NSCLC who developed TF had a history of esophageal toxicity caused by radiotherapy.

In this case, volumetric modulated arc therapy technique, enabling better radiation dose conformality to the target volume while simultaneously decreasing the dose to surrounding normal organs, was delivered. The mean esophageal dose was 26.0Gy, and the maximum esophageal dose was 62.2Gy, with an esophageal V50 of 32.4%. The mean dose in the location of later TF was 57.1Gy, the maximum dose was 61.8Gy, the minimum dose is 52.4Gy. And patient developed Grade I esophageal toxicity, Grade II gastrointestinal toxicity, Grade I dermatological toxicity and Grade I myelotoxicity during concurrent chemoradiotherapy.

TF is a rare but life-threatening complication of radiotherapy and bevacizumab in patients with NSCLC. Without early recognition and treatment, the patient would die of severe pulmonary infection. Gore et al. reported a case in which the unfortunate patient died of pneumonia caused by TF after the event.^[7]^

The commonly recommended treatment options for TF are jejunal feeding tube placement, esophageal stent, or surgery.^[11]^ In our case, due to timely diagnosis and treatment of esophageal stent, he recovered well in follow-up care more than 6 months after these events.

Careful observation is imperative for those patients who are administered bevacizumab, particularly in patients treated previously with radiotherapy. If patients present with a sudden onset of acute cough after drinking and they are suspected to have TF, then stent placement or surgery should be the treatment of choice.

## Acknowledgments

We thank the patient for allowing us to share this case and any accompanying images.

## Author contributions

**Conceptualization:** Tao Zhang, Nan Bi.

**Data curation:** Yin Yang, Ping Chen.

**Formal analysis:** Tao Zhang, Yin Yang.

**Resources:** Guowei Cheng.

**Writing – original draft:** Tao Zhang, Yin Yang.

**Writing – review and editing:** Tao Zhang, Nan Bi.

Tao Zhang orcid: 0000-0003-4861-0679.
